# High-resolution mapping of urban *Aedes aegypti* immature abundance through breeding site detection based on satellite and street view imagery

**DOI:** 10.1038/s41598-024-67914-w

**Published:** 2024-08-06

**Authors:** Steffen Knoblauch, Myat Su Yin, Krittin Chatrinan, Antonio Augusto de Aragão Rocha, Peter Haddawy, Filip Biljecki, Sven Lautenbach, Bernd Resch, Dorian Arifi, Thomas Jänisch, Ivonne Morales, Alexander Zipf

**Affiliations:** 1 GIScience Chair, Heidelberg University, 69120 Heidelberg, Germany; 2 Interdisciplinary Center of Scientific Computing, Heidelberg University, 69120 Heidelberg, Germany; 3 Heidelberg Institute for Geoinformation Technology, 69118 Heidelberg, Germany; 4 Faculty of ICT, Mahidol University, 73170 Nakhon Pathom, Thailand; 5 Institute of Computing, Fluminense Federal University, 24210-240 Niterói, Brazil; 6 Bremen Spatial Cognition Center, University of Bremen, 28359 Bremen, Germany; 7 Department of Architecture, National University of Singapore, 117566 Singapore, Singapore; 8 Department of Real Estate, National University of Singapore, 119245 Singapore, Singapore; 9 Geo-social Analytics Lab, Paris Lodron University of Salzburg, 5020 Salzburg, Austria; 10 Center for Geographic Analysis, Harvard University, 02138 Cambridge, USA; 11 Colorado School of Public Health, University of Colorado Anschutz Medical Campus, 80045 Aurora, USA; 12 Heidelberg Institute of Global Health, Heidelberg University Hospital, 69120 Heidelberg, Germany

**Keywords:** *Aedes aegypti*, Rio de Janeiro, Satellite, Street view, Object detection, Ovitrap, Risk factors, Ecological epidemiology

## Abstract

Identification of *Aedes aegypti* breeding hotspots is essential for the implementation of targeted vector control strategies and thus the prevention of several mosquito-borne diseases worldwide. Training computer vision models on satellite and street view imagery in the municipality of Rio de Janeiro, we analyzed the correlation between the density of common breeding grounds and *Aedes aegypti* infestation measured by ovitraps on a monthly basis between 2019 and 2022. Our findings emphasized the significance (*p* ≤ 0.05) of micro-habitat proxies generated through object detection, allowing to explain high spatial variance in urban abundance of *Aedes aegypti* immatures. Water tanks, non-mounted car tires, plastic bags, potted plants, and storm drains positively correlated with *Aedes aegypti* egg and larva counts considering a 1000 m mosquito flight range buffer around 2700 ovitrap locations, while dumpsters, small trash bins, and large trash bins exhibited a negative association. This complementary application of satellite and street view imagery opens the pathway for high-resolution interpolation of entomological surveillance data and has the potential to optimize vector control strategies. Consequently it supports the mitigation of emerging infectious diseases transmitted by *Aedes aegypti*, such as dengue, chikungunya, and Zika, which cause thousands of deaths each year.

## Introduction

The mosquito species *Aedes aegypti* is responsible for transmitting several communicable diseases, such as dengue, yellow fever, chikungunya, and Zika^1^. It has become an increasing global threat due to environmental changes associated with climate change, urban growth, and resistance to insecticides^2,3^. Dengue fever alone accounted for 390 million infections worldwide in 2020, marking a 30-fold increase over the last fifty years^4,5^. For this reason, numerous attempts have been made to enhance entomological surveillance methods for *Aedes aegypti* in order to predict patterns of potential disease outbreaks and conduct more targeted vector control^6,7^. However, the bioecology of *Aedes aegypti* turns the development of accurate monitoring techniques into a challenging task. *Aedes aegypti* is an urban favouring mosquito that breeds in small artificial water containers such as potted plants, and trash, which are often of ephemeral nature. This, combined with the bioecological assumption about a limited *Aedes aegypti* flight range of below 1000 m without the assistance of wind^8,9^, can result in a high spatial variability of abundance. High spatial variability is challenging to capture with traditional sample-based entomological field surveys^10^. The financial cost of such labor-intensive surveillance methods is also substantial, underscoring the urgent need for alternative mapping solutions, especially for urban areas of *Aedes aegypti*-endemic countries in the Global South where most infections occur^11^.

The increasing availability of openly accessible big spatial data, in combination with modern computing technologies, can help address these issues^12^. Digital techniques enable the large-scale interpolation of entomological surveillance data at a low financial cost. This enables the extrapolation of knowledge gathered from entomological sample locations into a continuous space, considering micro-scale changes in urban circumstances and the constrained flight range of mosquitoes. Consequently, these advancements could optimize the allocation of vector control resources, including more targeted spraying of insecticides and educational campaigns on local communities aimed at eliminating prevalent breeding sites^13–16^. The systematic reviews by Louis et al.^6^ and Sallam et al.^7^ summarized how satellite imagery and remote sensing techniques have successfully been applied in the past to estimate the spatial variance in *Aedes aegypti* abundance on both a global and local scale. They provided an extensive overview of hypothesis-driven indicators and modeling approaches that are instrumental in generating spatial suitability models for *Aedes aegypti*. However, both identified a gap in generating and evaluating urban indicators to capture *Aedes aegypti* distributions at mosquito flight range scale.

In this paper, we therefore propose a workflow of first generating high-resolution proxies to model *Aedes aegypti* abundance, and second, evaluating them with entomological surveillance data collected via ovitraps over a time period of four years. More precisely, we applied state-of-the-art computer vision models on satellite and street view imagery to detect common *Aedes aegypti* breeding sites. We chose these image datasets for their open accessibility, high resolution, and georeferencing, enabling a city-wide environmental analysis and the generation of urban micro-habitat indicators to estimate *Aedes aegypti* suitability at mosquito flight range scale. The joint application of both datasets promises to generate synergies. While both datasets have individually proven useful for this area of application in our previous studies^10,17,18^, their combined usefulness in this application field has not yet been investigated, to our knowledge. Therefore, our specific aim is to evaluate the following research question (RQ) to support future vector control of *Aedes aegypti*:

- **RQ:** Can the detection of *Aedes aegypti* breeding sites from satellite and street view imagery create statistically significant indicators (p ≤ 0.05) for modeling the abundance of *Aedes aegypti* immatures, measured by ovitraps, considering bioecological assumptions about a limited *Aedes* flight range (≤ 1000 m)?

## Methods

**Figure 1:**
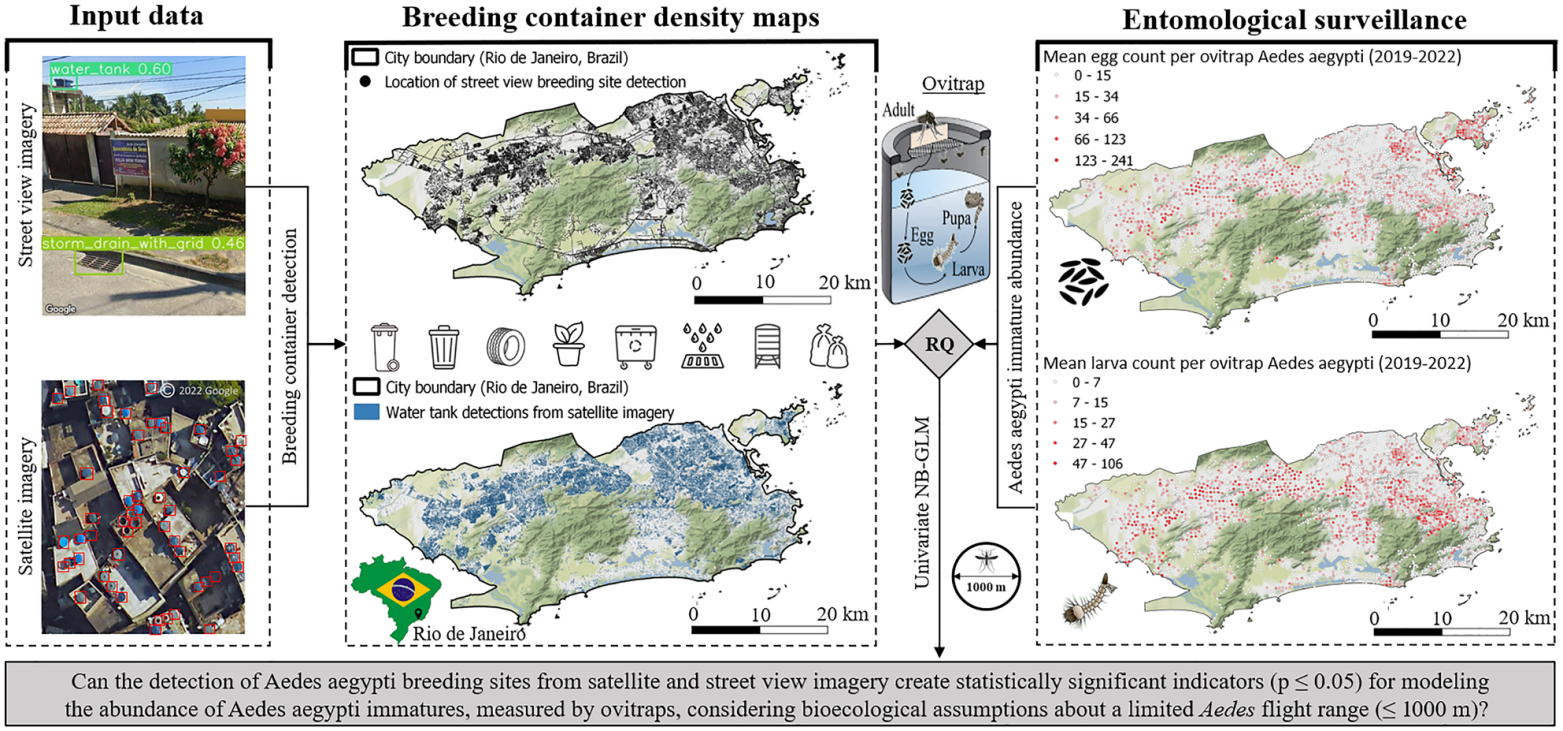
Workflow of evaluating the density of *Aedes aegypti* breeding container detections for modeling immature mosquito abundance at flight range scale in the city of Rio de Janeiro, Brazil. The mapping of *Aedes aegypti* breeding containers was carried out using satellite and street view imagery by applying and fine-tuning single-stage object detection networks (left). Container densities were calculated within a circular flight range buffer of 1000 m around ovitrap locations. For the evaluation of the research question, univariate negative binomial regression models were trained using temporally aggregated egg and larva counts from entomological surveillance (middle). Entomological surveillance data about immature abundance of *Aedes aegypti* was collected by the municipal health ministry of Rio de Janeiro (right). ©2023 Google.

Our experiment consists mainly of two parts (cf. Fig. 1): first, the detection of common breeding grounds for *Aedes aegypti* mosquitoes in urban areas, and second, the evaluation of container density for inference on urban *Aedes aegypti* abundance, considering limited mosquito flight range. We applied this workflow to the municipality of Rio de Janeiro, an endemic place for *Aedes aegypti* in Brazil, which is one of the worldwide hotspots for dengue, chikungunya, and Zika outbreaks^19,20^.

### Computer vision models for the detection of *Aedes aegypti* breeding habitat

The selection of breeding containers in this study was guided by a priori expectations derived from existing literature^21–29^, centered around their presumed influence on the abundance of *Aedes aegypti* immatures. The generation of water tank counts as a micro-habitat proxy derived from satellite imagery was extensively described in our previous research study^10^. In this previous work, we conceptualized a semi-supervised self-training algorithm to minimize the manual labeling effort for automated water tank detection in urban areas based on satellite imagery. We used a Single-Stage Object Detection network consisting of Inception-ResNet-V2 as a feature extractor and a multi-layer detector with a Non-Maximum Suppression layer pre-trained on the Microsoft COCO dataset^30^. We fine-tuned this model using 4000 manually labeled water tanks along with 10,400 pseudo water tank labels, encompassing various urban structure types, generated by the model during the training process. In our case, pseudo labels represented the results of model inference at 20,000 training iterations, applying a confidence threshold of 0.8. In total, the neural network was trained for 40,000 iterations: 20,000 initial iterations using manual labels only and 20,000 subsequent iterations using both manual and pseudo labels, which refers to a semi-supervised self-training procedure.

**Figure 2:**
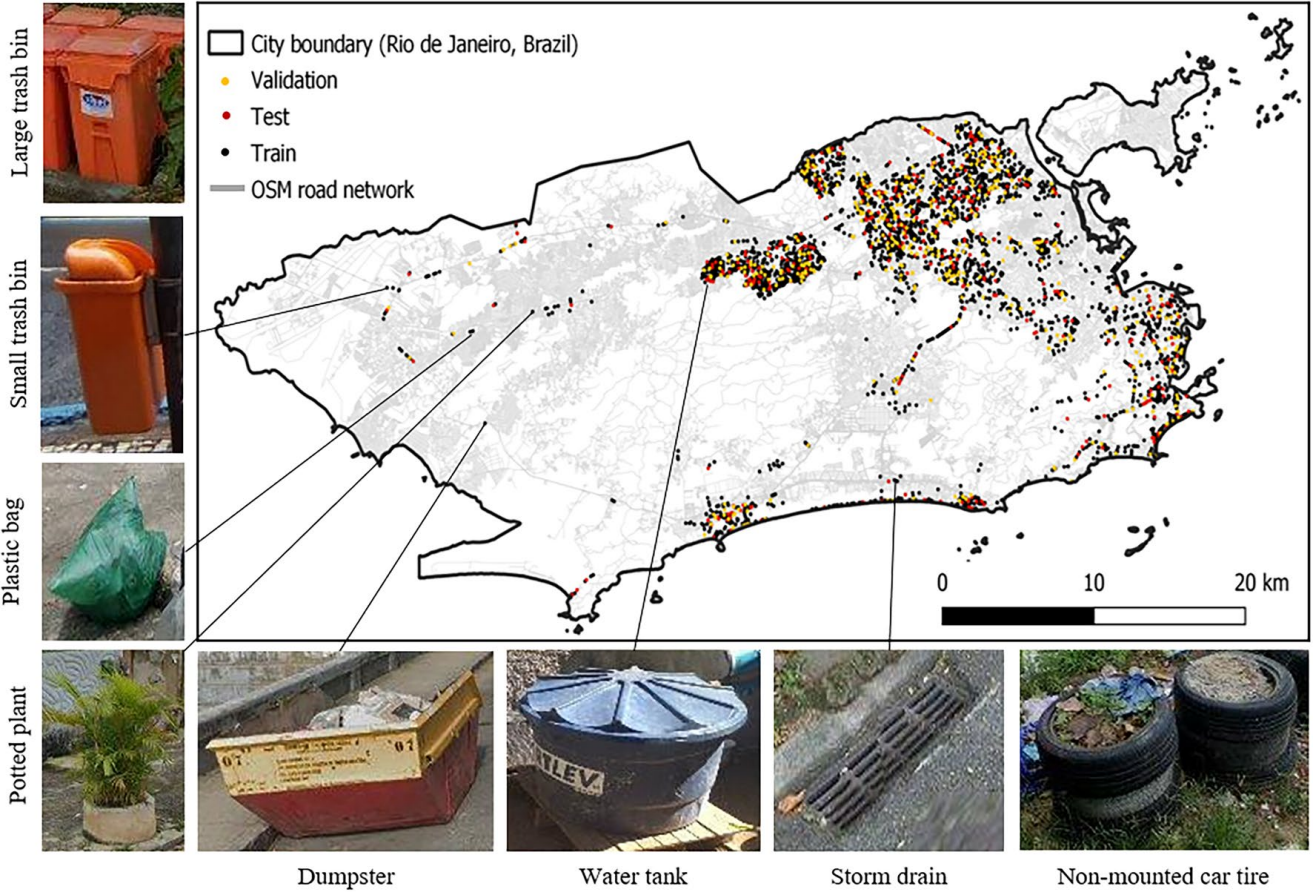
The images depicted identify breeding containers, accompanied by a map illustrating the coordinates of randomly selected train, test, and validation sets. These sets were chosen as subsets from a complete dataset of coordinates at 50 m intervals, encompassing the entire Open Street Map (OSM) road network in the municipality of Rio de Janeiro as of August 8th, 2023. Each train, test, and validation point corresponds to the downloading of five street view images, capturing a comprehensive 360-degree view at each location. This dataset compilation facilitated the training of object detection networks specifically tailored to identify *Aedes aegypti* breeding containers within the urban landscape. ©2023 Google.

In the present study, we additionally fine-tuned a multi-class object detector to map further *Aedes aegypti*-specific habitats as an extension of prior research. These habitats include potted plants, large and small trash bins, plastic bags, non-mounted car tires, water tanks, dumpsters, and storm drains (cf. Fig. 2). To detect these objects, we used street view images retrieved from Google’s Street View Static API^31^. A 50 m downloading interval for 360-degree street view images calculated from the OSM road network was deemed appropriate for the detection of mosquito breeding sites, following the approach used in other studies^17,18^. As of August 8th, 2023, this method yielded a total of 467,605 available street view images, which were utilized for labeling and city-wide container detection. The timestamps of the retrieved images ranged from January 2010 until 2023, with a share of 51% for images taken between 2022 and 2023, 15% from 2021, 19% from 2020 and 15% from before 2020. The downloaded image resolution was 600x500 pixels. For the supervised training of our multi-class object detector we manually labeled 7578 breeding containers on 3979 images using the graphical image annotation tool ‘labelImg’^32^. To minimize the manual labelling effort we implemented additional data augmentation techniques for instances of the ‘dumpster’ container class, which were observed infrequently within our dataset. We applied PCA color augmentation, horizontal flip and 180 degree rotation. The labelled dataset was then randomly divided into 80% for training, 10% for validation, and 10% for testing, resulting in 3152, 454, and 373 image subsets, respectively (cf. Table 1).

**Table 1:** Counts of images and labels for instances of *Aedes aegypti* breeding container detected in street view imagery, with differentiation between the train, validation, and test sets.

Dataset	Images	Labels	Instance							
			Dumpster	Large trash bin	Small trash bin	Non-mounted car tire	Plastic bag	Potted plant	Storm drain	Water tank
Train	3152	5729	310	428	400	625	757	1990	606	613
Validation	454	965	69	72	61	87	189	291	97	99
Test	373	884	67	60	57	129	173	197	81	120

**Figure 3:**
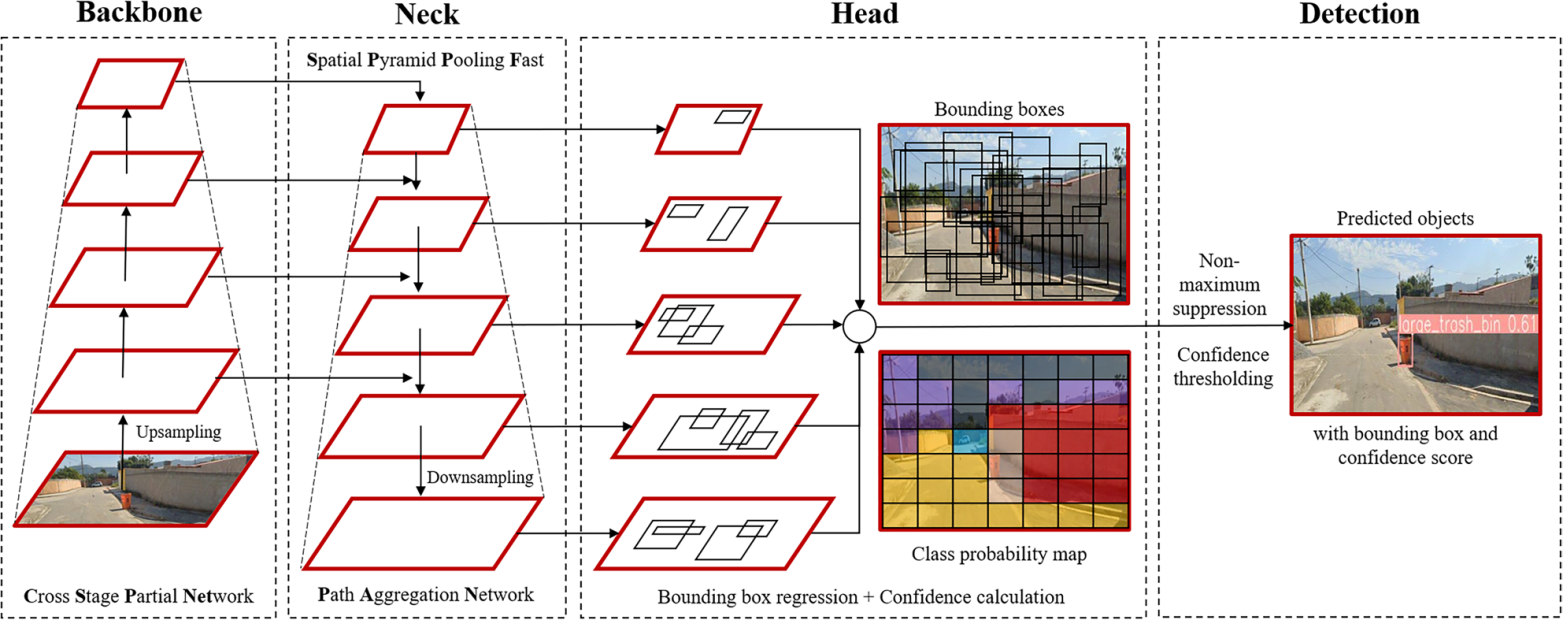
Schematic YOLOv5x architecture applying upsampling for semantic enrichment and downsampling to augment image resolution. The backbone component shaped feature maps at various levels of granularity. Subsequently, the neck module merged these feature maps and forwards them to the prediction head. In this stage, the features were utilized to perform precise box and class predictions. © 2023 Google.

Based on the street view imagery we fine-tuned a YOLOv5 model, which was pre-trained on the Microsoft COCO dataset^30^, specifically YOLOv5x, known for speed, accuracy, efficiency, adaptive architecture and scale-invariant detection (cf. Fig. 3). The applied model consisted of a CSPNet enhancing inter-layer information flow^33^, SPPF for multi-scale object analysis^34^, and PAN for parameter aggregation from different backbone levels^35^. During training, we fitted key parameters, utilizing AdamW for stability^36^. In three iterations of 300 epochs, we optimized the learning rate, adjusting it from $5e^{-5}$ to $1e^{-5}$, to enhance the efficiency of the model. Model convergence was reached after 900 epochs applying a patience parameter of 20. An iterative decrease of the focal loss parameter from 0.5 to 0.2 was implemented to cope with feature imbalance. Feature imbalance can lead to higher miss-classification rates for minority class instances^37^. The selected hyperparameters for training were listed in Appendix Table 5.

### Evaluation metrics for container detection and workflow of city-wide prediction

**Figure 4:**
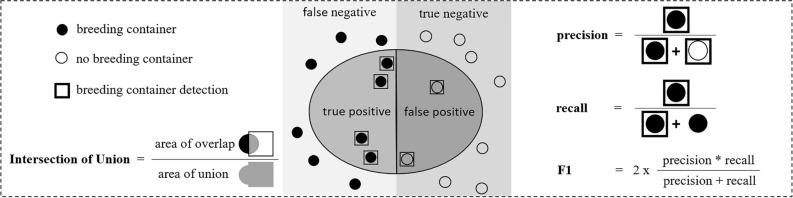
Schematic explanation of evaluation metrics applied to implemented *Aedes aegypti* breeding container detection networks.

Precision, recall, their harmonic mean, the F1-score, and the mean average precision at an Intersection over Union (IoU) threshold of 0.5 (mAP@0.5) were utilized to assess the object detection model performance (cf. Fig. 4). Precision is defined as the ratio of the true positive objects to all detected objects, while recall describes the fraction of relevant objects that are successfully retrieved. The performance metrics were computed based on the comparison between the intersection of the bounding boxes of the predictions and of the validation labels. This evaluation depended on the IoU value, which ranges between 0 and 1. An IoU Value of 0.5 or higher for a detected object was considered a true positive, while an IoU value lower than 0.5 indicated a false positive. Evaluated models were deployed to map the locations of *Aedes aegypti* breeding containers across the whole metropolitan area of Rio de Janeiro. In carrying out this task, street view images were processed in batches to sequentially predict bounding boxes and probabilities. Subsequent post-processing steps included non-maximum suppression and thresholding, with the application of a confidence score equal to or above 0.3. For the detection of water tanks in satellite imagery, we utilized over 10 million patches at zoom level 22 from the Bing Tile Map Service^38^. In this case, predictions were processed in parallel tasks. To organize the data, we employed the mapproxy API^39^, facilitating the storage of satellite imagery within a structured subset folder. Object detections for each image patch were pushed to a PostGIS database. The database was then used for a post-processing step to filter predictions with confidence scores of 0.7.

### Inference on *Aedes aegypti* immature abundance

To quantitatively evaluate the research question concerning how well the density of each detected mosquito breeding container can represent the spatial distribution of *Aedes aegypti* immatures within urban areas, as measured by entomological surveillance data, we ran univariate negative binomial generalized linear regression models (GLMs) employing log-link functions^40^. The selection of the negative binomial GLM was motivated by its capacity to account for the observed overdispersion in the entomological count data. For each of the nine detected breeding container types, two univariate models were conducted: one employing the ’mean egg per trap’ (MET) rate as the response variable (Mean = 19.8, Standard deviation = 20.12), and the other utilizing the ’mean larva per trap’ (MLT) rate as the response variable (Mean = 10.96, Standard deviation = 10.94). The associated mathematical formulations are delineated in Eq. 1. The entomological response variables $Y_i$ were averaged over 48 months of ovitrap surveillance ranging from January 2019 to December 2022. This was performed to yield robust spatial measurements over time, mitigating potential biases that could arise from the manual ovitrap collection process.

1 $$\begin{aligned} {Y}_i\sim & {} NB(\hat{\mu _i},{\hat{\theta }})\nonumber \\ {\mathbb {E}}({Y}_i)= & {} \hat{\mu _i} *(1-{\hat{\theta }})/{\hat{\theta }}\nonumber \\ \textrm{Var}({Y}_i)= & {} \hat{\mu _i} *(1-{\hat{\theta }})/{\hat{\theta }}^2\nonumber \\ \log (\hat{\mu _i})= & {} \hat{\beta _0} + \hat{\beta _1} *Breeding\;Container\;Count_i \end{aligned}$$

This approach led to the creation of two distinct models for each of the nine detected *Aedes aegypti* breeding containers. In these models, container density served as the independent variable, calculated using mosquito flight range buffers of 1000 m around ovitrap locations. For breeding containers identified through street view imagery, the counts were further normalized based on the number of retrieved images within each circular flight range buffer. This normalization accounted for observed variations in street coverage and image availability across different spatial locations. Our analysis incorporated data from a total of 2700 ovitrap locations, denoted by *i* in Eq. 1, each providing information on monthly egg and larva counts. The entomological data was provided upon request by the health ministry of the city of Rio de Janeiro. To scrutinize the robustness of our research findings concerning the estimated maximum flight ranges of *Aedes aegypti*, a sensitivity analysis was conducted by employing alternative buffer sizes of 250 and 500 m.

## Results and discussion

### Evaluation of breeding container detection

The multi-class object detection network trained on street view imagery achieved an overall F1 score of 0.878, indicating a balanced precision-recall trade-off (cf. Table 3). When examining specific classes, the breeding container class ‘dumpster’ showed the highest F1 score of 0.950, supported by a precision of 0.946 and a recall of 0.955. Similarly, the ‘large trash bin’ container type also has a high precision (0.933) and recall (0.930), contributing to an F1 score of 0.931. Although the ‘plastic bag’ container type demonstrates relatively lower precision (0.706), its recall (0.792) and F1 score (0.747) remain reasonable. On the other hand, the ‘potted plant’ container type achieves a high recall (0.959) alongside a corresponding F1 score of 0.865. The results for water tank detection in satellite imagery were extensively described in our previous research work^10^. In this previous research, the object detection model yielded a precision score of 0.864, a recall of 0.823, and an F1 score of 0.843 on independent test datasets.

**Table 2:** Goodness of fit indicators for the YOLOv5 model trained on street view imagery applying a confidence threshold of 0.3. The performance was based on independent test data points.

Breeding container	YOLOv5			
	Precision (%)	Recall (%)	F1	mAP@0.5
Water tank	0.801	0.867	0.833	0.895
Non-mounted car tire	0.876	0.837	0.856	0.901
Storm drain	0.884	0.941	0.912	0.955
Plastic bag	0.706	0.792	0.747	0.79
Potted plant	0.788	0.959	0.865	0.897
Large trash bin	0.933	0.93	0.931	0.959
Small trash bin	0.892	0.947	0.919	0.962
Large trash bin	0.933	0.93	0.931	0.959
Dumpster	0.946	0.955	0.950	0.98
Average weighted by instance	0.853	0.904	0.878	0.917

Interestingly, the ‘dumpster’ container class achieved the highest F1 score despite a smaller training set, necessitating augmentation techniques. Being larger containers with distinct characteristics might aid accurate identification in dumpsters. In contrast, the ‘plastic bag’ class recorded the lowest F1 score among all classes. This could be attributed to the inherent variability in plastic bag attributes like shape, size, and color. From 93,521 citywide coordinates, our model detected 2490 dumpsters, 7927 large trash bins, 6092 small trash bins, 24,034 non-mounted car tires, 43,334 plastic bags, 54,117 potted plants, 39,807 storm drains and 5898 water tanks from street view imagery.

**Figure 5:**
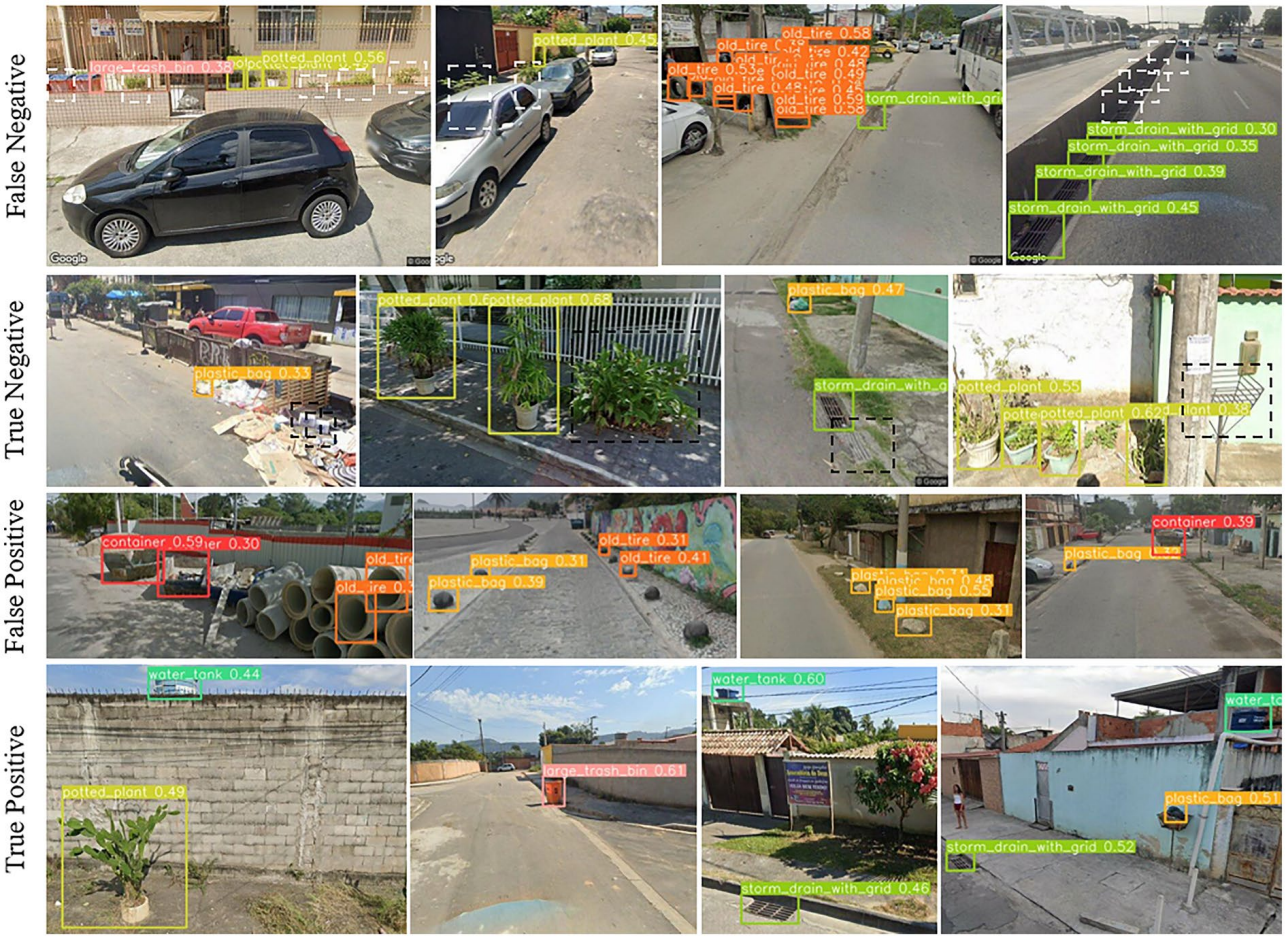
Example for False Negative, True Negative, False Positive, and True Positive breeding container predictions utilizing street view imagery. Detected breeding containers were indicated by bounding boxes, with distinct colors assigned to each container class. These visual representations were generated based on the confidence scores derived from a fine-tuned YOLOv5 model. In the two first image rows, white and black dashed bounding boxes were manually added to point to the locations of False Negative (white) and True Negative (black) examples, respectively, to enhance explanation. © 2023 Google.

Upon examining the results, we also identified several limitations. Detection of potted plants behind open fences was challenging as the fence’s pattern texture blends with the potted plant objects, leading to erroneous detection results (cf. Fig. 5). Vehicles parked along streets further complicated detection, potentially obstructing views of breeding containers. In detecting the water tank from the street view images, we observed that high-rise surroundings amplified the difficulty of identifying water tanks due to potential occlusions by neighboring structures. Other False Negative examples included sun-bleached water tanks, closely spaced storm drains, and overlaying containers such as plastic bags, water tanks, potted plants or non-mounted car tires. True Negative detections contained for example miscellaneous small containers in garbage heaps, plants without pots, sealed storm drains, and lattice trash bins. The most occurring False Positive cases included drainpipes detected as non-mounted car tires, truck loads detected as dumpsters and larger stones used as road boundaries falsely detected as either plastic bags or non-mounted car tires. An object class labeled as ‘miscellaneous small containers’, representing for example trash piles, was excluded during the training phase due to the absence of clear 3D object features and its varied appearance. These characteristics made it challenging to capture this potential breeding site using our object detection network for street view imagery. An additional training of scene recognition model could potentially address these limitations associated with this object class.

In our previous research work on water tank detection in satellite imagery^10^, common False Negative predictions included water tanks in shaded or partially shaded areas. To mitigate the occurrence of false negative predictions, one could enhance the precision of the water tank detection network by augmenting its training data with additional instances of shaded water tank labels. It is important to highlight that our study area contained a substantial number of objects resembling water tanks, leading to a notable prevalence of False Positives. While our models rarely misclassified similar objects like blue cars and rooftop ventilators as water tanks, circular water pools and blue sunshades on beaches consistently resulted in False Positives. Addressing the False Positive detections of water pools could involve the implementation of a size filter, while False Positives associated with blue sunshades on beaches could be alleviated through the application of an automatic land use map-based filter. However, it is crucial to recognize that these solutions have inherent limitations. Specifically, they may not be effective for very small water pools and blue sunshades not situated on beaches, rendering the proposed methods obsolete. An alternative approach would involve filtering predictions based on confidence scores.

**Figure 6:**
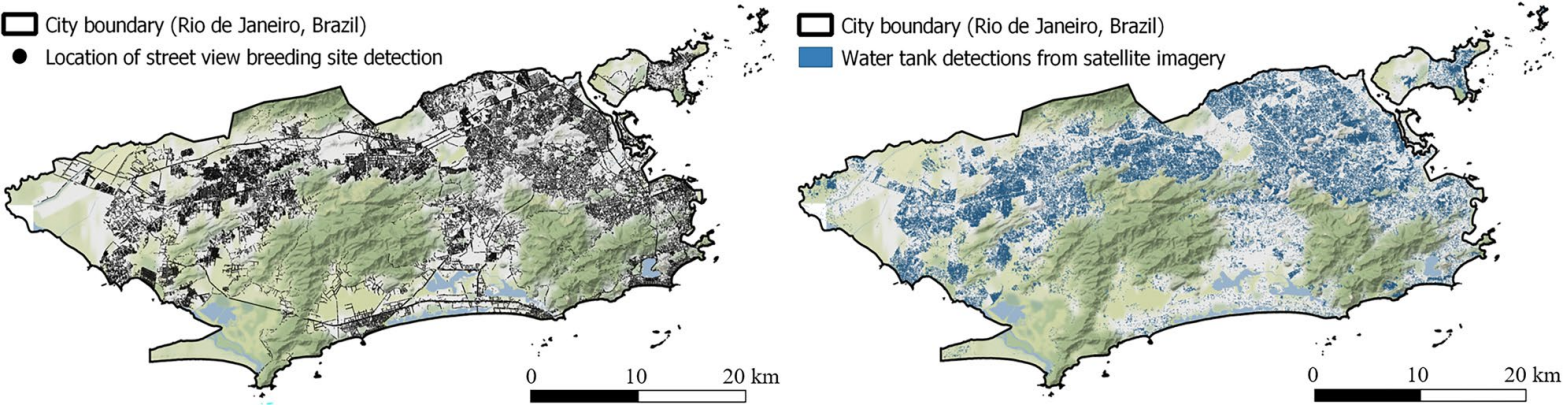
Large-scale *Aedes aegypti* breeding site detection from 461,152 street view and satellite imagery for the municipality of Rio de Janeiro, Brazil. Left map shows location of retrieved street view images used for 360-degree breeding site detection and right maps highlights water tank density detected from satellite imagery generated in Knoblauch et al.^10^.

As a highlight of the present research work, we created density maps of *Aedes aegypti* breeding containers detected over the entire municipal area of Rio de Janeiro using the combined dataset of satellite and street view imagery (cf. Fig. 6). All detected breeding containers exhibited a widespread distribution across the study region, characterized by substantial spatial variation. Satellite imagery played a crucial role in detecting breeding containers located in residential backyards and on top of buildings, while street view imagery complementary identified such containers on streets, beneath trees, and within sheltered areas (cf. Table 3). While the detection of mosquito breeding sites in satellite imagery was limited to water tanks due to the image resolution of 0.0373 m per pixel^10^, street view images enabled the detection of even small breeding containers like plastic bags. However, satellite imagery has the advantage of providing continuous spatial coverage. The availability of street view images within the road network of the municipality of Rio de Janeiro was restricted, especially in narrow and impassable streets that are common in favelas. Generally, the prevalence of artificial mosquito breeding containers was strongly linked to inhabited regions, with forested areas notably lacking such containers.

**Table 3:** Qualitative attribute comparison of satellite and street view imagery, underscoring the essential need for a complementary application of these digital sources when mapping *Aedes aegypti* breeding sites.

Attribute	Street view imagery	Satellite imagery
Spatial coverage	Limited coverage defined by visited road network.	Complete coverage.
	Ground-level perspective below shelters and tree canopy.	Monitoring of inaccessible backyards and rooftops.
Image resolution	Detection of small and large breeding container.	Fails at capturing smaller breeding sites.
Open-accessibility	Open-accessibility with monthly downloading limit.	Open-accessibility at limited resolution.
	Open-source alternatives without limits.	Commercial high-resolution alternatives.
Temporal updates	Infrequent, non-collective updates of images.	Infrequent, collective updates of all images.
Provider	Google^31^/ Mapilio^41^/ Mapillary^42^/ KartaView^43^/...^44^	Microsoft^38^/ NASA^45^/ Copernicus^46^/ Planet^47^/.

### Modeling of immature *Aedes aegypti* infestation

The results of our negative binomial linear regression models (cf. Table 4) indicated that all detected breeding containers of this study were highly significant (*p* ≤ 0.05) proxies for modeling urban *Aedes aegypti* immature abundance while considering limited mosquito flight range below 1000 m. This was in line with our expectations and implies that breeding site density maps can be a useful indicator to enrich entomological surveillance data and thus support future vector control by providing more continuous and high resolution insights for urban mosquito distributions.

**Table 4:** Coefficients, standard errors, and *p*-values for univariate negative binomial generalized linear regression models applying a *Aedes* flight range buffer of 1000 m around 2700 ovitrap locations. Regression coefficients and standard errors were reported at the link scale. The p-value of the intercept was $< 2e^{-16}$ for all models. (Water tank* = Water tanks detected with satellite imagery).

Breeding container	Response	Intercept		Breeding Container			Theta		Metrics	
		Estimate	Std. Error	Estimate	Std. Error	*p* value	Estimate	Std. Error	Pseudo R2	AIC
Water tank*	MET	2.8493	0.0296	0.0026	0.0004	$2.03e^{-09}$	1.1990	0.0363	0.0123	17645
	MLT	2.1046	0.0298	0.0053	0.0004	$< 2e^{-16}$	1.2937	0.0435	0.0496	14551
Water tank	MET	2.8448	0.0272	0.2580	0.0358	$5.49e^{-13}$	1.2045	0.0365	0.0172	17634
	MLT	2.2253	0.0280	0.3077	0.0369	$< 2e^{-16}$	1.2505	0.0417	0.0225	14620
Non-mounted car tire	MET	2.9169	0.0230	0.2872	0.0505	$1.26e^{-08}$	1.1964	0.0362	0.0110	17650
	MLT	2.2941	0.0234	0.4027	0.0506	$1.78e^{-15}$	1.2506	0.0417	0.0223	14620
Storm drain	MET	2.8028	0.0431	0.4343	0.0926	$2.7e^{-06}$	1.1927	0.0361	0.0082	17657
	MLT	2.1101	0.0441	0.6704	0.0945	$1.29e^{-12}$	1.2431	0.0414	0.0176	14632
Plastic bag	MET	2.8694	0.0316	0.2583	0.0558	$3.7e^{-06}$	1.1909	0.0360	0.0068	17660
	MLT	2.1996	0.0324	0.4223	0.0566	$8.44e^{-14}$	1.2436	0.0414	0.0179	14631
Potted plant	MET	2.8723	0.0328	0.1870	0.0440	$2.11e^{-05}$	1.1908	0.0360	0.0067	17661
	MLT	2.2224	0.0336	0.2837	0.0454	$4.03e^{-10}$	1.2369	0.0411	0.0134	14642
Small trash bin	MET	3.1111	0.0245	-1.6963	0.1775	$<2e^{-16}$	1.2178	0.0370	0.0276	17607
	MLT	2.4966	0.0252	-1.3805	0.1865	$1.32e^{-13}$	1.2428	0.0414	0.0177	14632
Large trash bin	MET	3.0765	0.0270	-1.0363	0.1953	$1.12e^{-07}$	1.1958	0.0362	0.0105	17651
	MLT	2.4863	0.0278	-1.0553	0.2063	$3.13e^{-07}$	1.2325	0.0410	0.0102	14650
Dumpster	MET	3.0561	0.0243	-2.4497	0.4461	$4e^{-08}$	1.1973	0.0363	0.0116	17648
	MLT	2.4648	0.0249	-2.4589	0.4636	$1.13e^{-07}$	1.2340	0.0410	0.0112	14648

Water tanks, non-mounted car tires, storm drains, plastic bags, and potted plants consistently displayed positive coefficient estimates for both response variables, whereas the coefficient estimates for small and large trash bins, as well as dumpsters, consistently demonstrated negativity across both model variations. These findings aligned with the intuitive understanding that an increased presence of trash bins of any kind correlates with a reduced prevalence of uncontained refuse piles, thereby mitigating the potential for additional mosquito breeding sites. The correlation between the density of plastic bags and all three trash container classes was found to be negative, namely − 0.1 for the dumpster class, − 0.03 for large trash bins, and − 0.3 for small trash bins. In addition, small and large trash bins, as well as dumpsters, are usually closed containers that rarely fill with water when it rains, which underlines their significance (*p* ≤ 0.05) and negative association with entomological data about *Aedes aegypti* immature abundance. Furthermore, these containers are regularly emptied by refuse collection services, ensuring that they often remain dry and unsuitable for mosquito breeding, thus contributing to mosquito control efforts.

When analyzing the results independently from the response variable, it was observed that models using water tank density derived from satellite and street view imagery consistently led to the lowest Akaike information criterion (AIC), indicating a superior fit to the data across both immature abundance stages. Conversely, models employing the density of potted plants displayed the highest AIC values in relation to the MET rate, while models utilizing the density of large trash bins exhibited the highest AIC values in relation to the MLT rate. The extent of explained deviance in the regression models pertaining to the MLT rate generally exhibited higher values compared to those associated with the MET rate. Specifically, the MLT model, utilizing water tank density derived from satellite imagery, achieved the highest explained deviance at 0.05 as quantified by Cohen’s pseudo-$\text {R}^2$^48^ (cf. Eq. 2). This indicates that approximately 5% of the variance in the response variable is accounted for by the univariate model.

2 $$\begin{aligned} Cohen's\ pseudo\ R^2= & {} 1 - \frac{model\ deviance}{null\ model\ deviance}\nonumber \\ Negative\ binomial\ model\ deviance= & {} 2\sum \left( y \cdot log\left( \frac{y}{\mu }\right) - (y+k^-1)log\left( \frac{y+k^-1}{\mu +k^-1}\right) \right) \end{aligned}$$

The deviance function of the negative binomial GLM captured the increasing variance with the mean that is typical for count data. The dispersion parameter captures how much the variance increases with the mean relative to a Poisson GLM, where the variance equals the mean. The theta values of all univariate regression models in this study indicated a sustantial overdispersion. This overdispersion can be attributed to two primary factors. First, the dataset on entomological observations contained a substantial number of zero values, necessitating the adoption of a negative binomial GLM to account for excess variation. Second, the limited inclusion of predictors in modeling the urban distribution of *Aedes aegypti* also contributed to the observed low value of explained deviance. It is worth noting that certain potentially relevant predictors have been intentionally omitted from the model, further contributing to the constrained explanatory power. The incorporation of additional explanatory variables is planned for subsequent phases of this research.

The outcomes of the performed sensitivity analysis (cf. Appendix Table 6), scrutinizing different assumed maximum flight ranges of *Aedes aegypti* (250 m, 500 m, 1000 m), confirmed the robustness of the results outlined in Table 4. Similar to the results for a 1000 m *Aedes aegypti* maximum flight range, at a maximum flight range of 500 m, all container types exhibited significant *p* values (*p* ≤ 0.05) for both egg and larva counts. The same trend was observed for the assumed maximum *Aedes aegypti* flight range of 250 m, except for the container types dumpster, storm drain, and water tank detected from satellite imagery. Notably, the findings concerning water tanks from satellite imagery at 250 m scale show a slight contrast to our previous findings in Ref^10^, where a different time frame for entomological data was utilized; however, significance was detected at a flight range scale of 200 m. This divergence of these findings underscores the considerable influence of the selected time period of entomological surveillance on the validation of such results. The coefficients for small and large trash bins, as well as the dumpster category, remained negative also at lower estimated maximum *Aedes aegypti* flight ranges. Intriguingly, the coefficient for potted plants shifted from positive to negative when simulating a maximum flight range of 250 m for *Aedes aegypti*. Overall, there was an evident upward trend in significance (indicated by a downward trend in *p*-values) across all container classes, with larger buffer sizes, representing simulations of larger flight ranges, showing higher significance levels. Essentially, larger buffer areas augment the probability of encountering containers, consequently yielding more dependable statistical outcomes in our methodology for modeling ovitrap count data with digital proxies. For a more nuanced understanding of the relationship between assumed maximum *Aedes aegypti* flight range and significance values, models implementing soft constraints could be considered, such as Bayesian models.

The collective findings presented in this study offer a comprehensive overview and extension of our prior research about urban mosquito mapping^10,17,18^. For the first time the results underscore the practical efficacy of integrating satellite and street view imagery for identifying mosquito breeding sites in urban areas, emphasizing the distinctive advantages of each method. A further alternative data source for mapping mosquito breeding containers in urban areas could be drone imagery, which offers both continuous spatial coverage and images in high resolution for small breeding container detection^49,50^. However, it is essential to note that generating drone imagery incurs substantial costs and labor, thereby limiting the applicability in diverse global urban settings. A common limitation across all three data sources is their inability to detect breeding containers located inside buildings. Consequently, the digital strategies outlined in this study cannot fully replace on-site entomological surveillance. Instead, our approach aims to complement manual monitoring efforts by augmenting them with high-resolution digital information. Citizen Science offers a promising avenue to address this limitation, fostering public participation, including crowdsourced mapping, to enhance data collection and monitoring, particularly of indoor breeding sites. The primary challenge in utilizing digital data sources for mosquito mapping lies in achieving temporal alignment with entomological surveillance for modeling purposes.

Another challenge associated with digital data sources, such as satellite and street view imagery, pertains to the potential obsolescence of information and the insights derived from it. Street view images, in particular, are infrequently updated^51^. It is also crucial to consider the transient nature and shifting locations of identified containers, especially for plastic bags, potted plants, non-mounted car tires, large trash bins, and dumpsters, which may have introduced a potential bias to the measured significance values of these container classes in our results. Conversely, water tanks, small city trash bins attached to streetlights, and storm drains are presumed to have relatively stable locations over time, leading to more reliable results. Furthermore, the calculated container densities in this study may be influenced by citywide solid waste collections or vector control campaigns, wherein breeding containers may have been removed before images were captured. In future studies, investigating the relationship between image timestamps and such interventions, as well as exploring alternative data sources (cf. Table 3), could be beneficial. Crowd-sourced platforms such as Mapillary^42^ and KartaView^43^ may particularly offer more continuous image updates^52^.

In summary, this study demonstrated the enhanced efficiency in managing urban diseases such as dengue through the application of digital techniques. The increasing availability of spatial big data, such as satellite and street view imagery, presents a considerable opportunity for obtaining high-resolution indicators for mapping urban mosquito suitability beyond entomological sample points and allows interpolations without violating biological assumptions about limited mosquito flight ranges in the future. The proposed approach can be combined with further urban-specific mosquito proxies for enabling more targeted vector control. A task that is challenging with entomological surveillance alone. The proposed method can thus not only reduce surveillance costs but also facilitates the potential interruption of infection chains at earlier stages of an outbreak than with conventional methods.

## Data Availability

The datasets generated and analyzed during the current study are available from the corresponding author on reasonable request. Restrictions apply only to the sharing of entomological surveillance data collected by the Municipal Health Ministry of Rio de Janeiro.

## Appendix

See Tables 5 and 6

**Table 5:** Hyperparameters used for training. The training and detection processes were conducted using Google Colab, a cloud computing platform offering 53 GB of Random Access Memory (RAM), 8 CPU cores, a Tesla V100 GPU, and 150 GB of disk space.

Batch size	24
Learning rate	0.0004
IoU threshold	0.5
Optimizer	RMSprop
Optimizer momentum	0.9
Optimizer decay	0.9
Optimizer epsilon	0.1

**Table 6:** Coefficients, standard errors, and p-values for univariate negative binomial generalized linear regression models applying a mosquito flight range buffer of 250 and 500 m around 2700 ovitrap locations. Regression coefficients and standard errors were reported at the link scale. The p-value of the intercept was $2e^{-16}$ for all models. (Water tank* = Water tanks detected with satellite imagery).

Breeding container	Response	Modelled flight range (m)	Intercept		Breeding container			Theta		Metrics	
			Estimate	Std. Error	Estimate	Std. Error	*p*-value	Estimate	Std. Error	Pseudo R2	AIC
Water tank*	MET	$\le$250	2.9734	0.0256	0.0013	0.0018	0.4450	1.1824	0.0357	0.0002	17,677.0000
		$\le$500	2.9655	0.0229	0.0085	0.0047	0.0410	1.1831	0.0358	0.0008	17,676.0000
	MLT	$\le$250	2.3954	0.0261	-0.0001	0.0018	0.9510	1.2172	0.0403	$1.304e^{-6}$	14,675.0000
		$\le$500	2.3312	0.0234	0.0258	0.0047	$3.43e^{-8}$	1.2262	0.0407	0.0063	14,660.0000
Water tank	MET	$\le$250	2.9450	0.0235	0.0839	0.0262	0.0014	1.1874	0.0359	0.0041	17,667.0000
		$\le$500	2.8928	0.0252	0.1785	0.0312	$1.05e^{-08}$	1.1971	0.0363	0.0116	17,648.0000
	MLT	$\le$250	2.3576	0.0241	0.0759	0.0267	0.0045	1.2219	0.0405	0.0032	14,667.0000
		$\le$500	2.2799	0.0259	0.2182	0.0321	$1e^{-11}$	1.2411	0.0413	0.0161	14,635.0000
Non-mounted car tire	MET	$\le$250	2.9732	0.0205	0.0606	0.0223	0.0066	1.1871	0.0359	0.0037	17,668.0000
		$\le$500	2.9599	0.0211	0.1163	0.0311	0.0002	1.1883	0.0360	0.0048	17,666.0000
	MLT	$\le$250	2.3840	0.0210	0.0510	0.0224	0.0229	1.2215	0.0405	0.0028	14,668.0000
		$\le$500	2.3609	0.0215	0.1494	0.0314	$1.96e^{-6}$	1.2288	0.0408	0.0079	14,656.0000
Storm drain	MET	$\le$250	2.9883	0.0252	-0.0078	0.0419	0.8530	1.1822	0.0357	$1.47e^{-5}$	17,678.0000
		$\le$500	2.9069	0.0309	0.1916	0.0583	0.0010	1.1869	0.0359	0.0037	17,668.0000
	MLT	$\le$250	2.4173	0.0259	-0.0655	0.0438	0.1350	1.2186	0.0404	0.0010	14,673.0000
		$\le$500	2.2785	0.0316	0.2797	0.0593	$2.44e^{-6}$	1.2277	0.0408	0.0072	14,657.0000
Plastic bag	MET	$\le$250m	2.9626	0.0228	0.0565	0.0272	0.0375	1.1844	0.0358	0.0017	17,673.0000
		$\le$500	2.9524	0.0249	0.0754	0.0342	0.0273	1.1842	0.0358	0.0016	17,674.0000
	MLT	$\le$250	2.3757	0.0234	0.0465	0.0282	0.0982	1.2189	0.0404	0.0011	14,672.0000
		$\le$500	2.3381	0.0255	0.1254	0.0346	0.0003	1.2234	0.0406	0.0042	14,665.0000
Potted plant	MET	$\le$250	3.0241	0.0238	-0.0796	0.0253	0.0016	1.1869	0.0359	0.0037	17,668.0000
		$\le$500	2.9210	0.0270	0.1058	0.0305	0.0005	1.1878	0.0359	0.0043	17,667.0000
	MLT	$\le$250	2.4277	0.0245	-0.0687	0.0262	0.0088	1.2211	0.0405	0.0027	14,669.0000
		$\le$500	2.3076	0.0277	0.1420	0.0314	$5.97e^{-6}$	1.2277	0.0408	0.0072	14,658.0000
Small trash bin	MET	$\le$250	3.0202	0.0210	-0.6147	0.0963	1.77 e-10	1.2039	0.0365	0.0164	17,636.0000
		$\le$500	3.0274	0.0227	-0.5604	0.1320	$2.18e^{-5}$	1.1907	0.0360	0.0067	17,661.0000
	MLT	$\le$250	2.4299	0.0216	-0.6224	0.1005	$5.95e^{-10}$	1.2415	0.0414	0.0159	14,636.0000
		$\le$500	2.4275	0.0233	-0.4449	0.1383	0.0013	1.2227	0.0406	0.0038	14,666.0000
Large trash bin	MET	$\le$250	3.0038	0.0211	-0.2511	0.0788	0.0014	1.1872	0.0359	0.0039	17,668.0000
		$\le$500	3.0058	0.0227	-0.2305	0.1155	0.0459	1.1842	0.0358	0.0015	17,674.0000
	MLT	$\le$250	2.4106	0.0216	-0.2168	0.0806	0.0072	1.2212	0.0405	0.0027	14,669.0000
		$\le$500	2.4224	0.0233	-0.3204	0.1192	0.0072	1.2212	0.0405	0.0027	14,669.0000
Dumpster	MET	$\le$250	2.9850	0.0205	0.0161	0.1230	0.8960	1.1822	0.0357	$9.98e^{-6}$	17,678.0000
		$\le$500	3.0061	0.0214	-0.6987	0.2279	0.0022	1.1871	0.0359	0.0038	17,668.0000
	MLT	$\le$250	2.3990	0.0210	-0.1589	0.1290	0.2180	1.2185	0.0404	0.0008	14,673.0000
		$\le$500	2.4179	0.0219	-0.8032	0.2349	0.0006	1.2241	0.0406	0.0047	14,664.0000
