# Author Correction: High-resolution mapping of urban *Aedes aegypti* immature abundance through breeding site detection based on satellite and street view imagery

**DOI:** 10.1038/s41598-024-73687-z

**Published:** 2024-10-04

**Authors:** Stefen Knoblauch, Myat SuYin, Krittin Chatrinan, Antonio Augusto de Aragão Rocha, Peter Haddawy, Filip Biljecki, Sven Lautenbach, Bernd Resch, Dorian Arif, Thomas Jänisch, Ivonne Morales, Alexander Zipf

**Affiliations:** 1 GIScience Chair, Heidelberg University, 69120 Heidelberg, Germany; 2 Interdisciplinary Center of Scientifc Computing, Heidelberg University, 69120 Heidelberg, Germany; 3 Heidelberg Institute for Geoinformation Technology, 69118 Heidelberg, Germany; 4 Faculty of ICT, Mahidol University, Nakhon Pathom, 73170 Thailand; 5 Institute of Computing, Fluminense Federal University, Niterói, 24210‑240 Brazil; 6 Bremen Spatial Cognition Center, University of Bremen, 28359 Bremen, Germany; 7 Department of Architecture, National University of Singapore, Singapore, 117566 Singapore; 8 Department of Real Estate, National University of Singapore, Singapore, 119245 Singapore; 9 Geo‑social Analytics Lab, Paris Lodron University of Salzburg, 5020 Salzburg, Austria; 10 Center for Geographic Analysis, Harvard University, Cambridge, 02138 USA; 11 Colorado School of Public Health, University of Colorado Anschutz Medical Campus, Aurora, 80045 USA; 12 Heidelberg Institute of Global Health, Heidelberg University Hospital, 69120 Heidelberg, Germany

Correction to: *Scientific Reports*10.1038/s41598-024-67914-w, published online 06 August 2024

The original version of this Article contained an error in Reference 10, which was incorrectly given as:

Knoblauch, S. *et al.* High-resolution mapping of urban aedes aegypti immature abundance through breeding site detection based on satellite and street view imagery (under review) *Scientifc Reports: Collection on Urbanization and communicable diseases*, (2023).

The correct reference is listed below:

Knoblauch, S. et al. Semi-supervised water tank detection to support vector control of emerging infectious diseases transmitted by *Aedes Aegypti. International Journal of Applied Earth Observation and Geoinformation*, **119,**10.1016/j.jag.2023.103304 (2023).

The original Article has been corrected.

